# Identifying Advanced Biotechnologies to Generate Biofertilizers and Biofuels From the World’s Worst Aquatic Weed

**DOI:** 10.3389/fbioe.2021.769366

**Published:** 2021-12-22

**Authors:** Amine Ezzariai, Mohamed Hafidi, Widad Ben Bakrim, Mulugeta Kibret, Fadoua Karouach, Mansour Sobeh, Lamfeddal Kouisni

**Affiliations:** ^1^ African Sustainable Agriculture Research Institute, Mohammed VI Polytechnic University, Laayoune, Morocco; ^2^ Laboratoire Biotechnologies Microbiennes, Agrosciences et Environnement (BioMagE), Unité de Recherche Labellisée, Faculty of Science Semlalia, Cadi Ayyad University, Marrakech, Morocco; ^3^ Agrobiosciences Department, Mohammed VI Polytechnic University, Benguérir, Morocco; ^4^ Department of Biology, Bahir Dar University, Bahir Dar, Ethiopia

**Keywords:** water hyacinth, composting, anaerobic digestion, methane, hydrogen, ethanol, biofuels

## Abstract

Water hyacinth (*Eichhornia crassipes L*.) was introduced as an invasive plant in freshwater bodies more particularly in Asia and Africa. This invasive plant grows rapidly and then occupies a huge layer of freshwater bodies. Hence, challenges are facing many countries for implementing suitable approaches for the valorization of the world’s worst aquatic weed, and water hyacinth (WH). A critical and up-to-date review article has been conducted for more than 1 year, based on more than 100 scientific journal articles, case studies, and other scientific reports. Worldwide distribution of WH and the associated social, economic, and environmental impacts were described. In addition, an extensive evaluation of the most widely used and innovative valorization biotechnologies, leading to the production of biofertilizer and bioenergy from WH, and was dressed. Furthermore, an integrated search was used in order to examine the related advantages and drawbacks of each bioprocess, and future perspectives stated. Aerobic and anaerobic processes have their specific basic parameters, ensuring their standard performances. Composting was mostly used even at a large scale, for producing biofertilizers from WH. Nevertheless, this review explored some critical points to better optimize the conditions (presence of pollutants, inoculation, and duration) of composting. WH has a high potential for biofuel production, especially by implementing several pretreatment approaches. This review highlighted the combined pretreatment (physical-chemical-biological) as a promising approach to increase biofuel production. WH valorization must be in large quantities to tackle its fast proliferation and to ensure the generation of bio-based products with significant revenue. So, a road map for future researches and applications based on an advanced statistical study was conducted. Several recommendations were explored in terms of the choice of co-substrates, initial basic parameters, and pretreatment conditions and all crucial conditions for the production of biofuels from WH. These recommendations will be of a great interest to generate biofertilizers and bioenergy from WH, especially within the framework of a circular economy.

**GRAPHICAL ABSTRACT:**
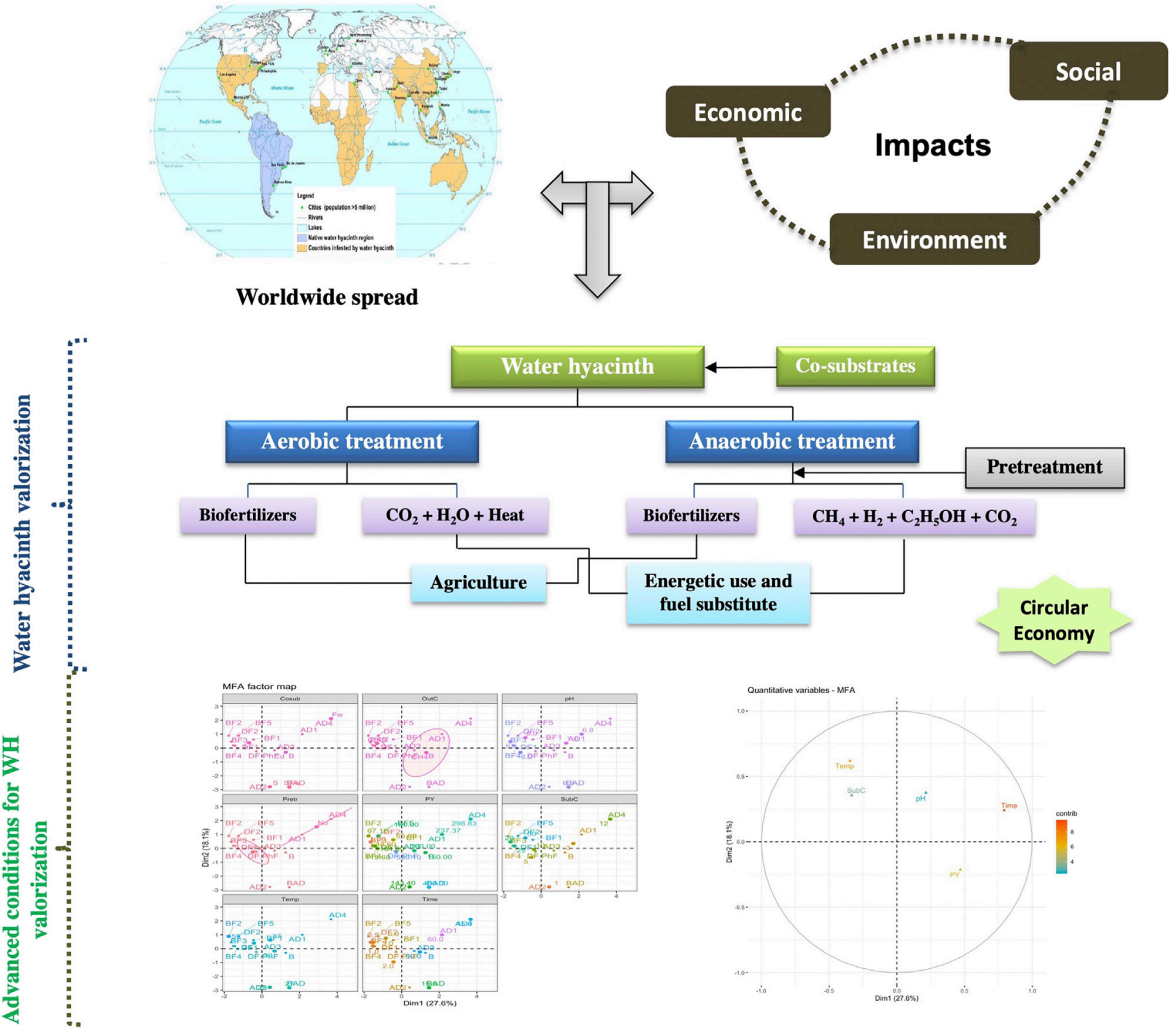


## 1 Highlight

⁃ Water hyacinth spread is a challenging social, economic and the environmental problem
⁃ Composting is recommended for water hyacinth valorization at large-scale
⁃ Combined pretreatment is a promising approach for biofuel generation
⁃ Commercialization of bioenergy from water hyacinth still need more efforts

## 2 Introduction

Native to the Amazon River Basin in South America, WH was discovered by a German naturalist, and von Martiusin 1823 (Téllez et al., 2008). Since the end of the 19th century, this invasive plant has spread from its natural habitat to a large area in the tropical and subtropical regions of the world (North America, Asia, Australia, and Africa). The International Union for Conservation of Nature classified WH as one of the 100 most aggressive invasive species and recognized as one of the worst weeds in the world (Gichuki et al., 2012; Patel, 2012). WH is considered as fastest growing plant in aquatic ecosystem. For examples, it was reported that the infestation area in Lake Tana (Ethiopia) is up to 50,000 ha, and with an expansion rate of about 13 ha/day (Dersseh et al., 2019; Yitbarek et al., 2019).

WH spread is a major challenge causing social, economic, and environmental impacts. The proliferation of this invasive plant clogs irrigation canals, drinking water, and electricity production as well as fishing activities. In addition, In addition, WH blocks light penetration and then induces changes in the functions of aquatic ecosystems (Dersseh et al., 2019). Through all these modifications, local microorganisms, plants, and animals could lose their original habitats. Several methods (physical, chemical, and biological) were implemented to remove and control WH proliferation. The physical method involves machines or manual removal by hands and/or hand tools and is mainly adapted to small lakes due to its high costs and huge logistics, equipment, and labor force needs. Herbicides and pesticides were also applied as a chemical method to eradicate or reduce the level of WH growth (Admas et al., 2017; Worqlul et al., 2020). However, these chemicals have ecological and adverse side effects and they are not recommended when using water for drinking purposes. Some biological methods are also adopted through using insects and fungi (Hill and Coetzee, 2008), although the potential adaptability of these species must be first investigated. Large quantities of WH are required for using one of the aforementioned listed control methods. So, high economic concern could be created through producing significant amount of biofertilizers and bioenergy.

WH is a lignocellulosic biomass with high potential for bioconversion to biofertilizers and biofuels, based onadvanced biotechnologies (Li et al., 2021; Nawaj Alam et al., 2021; Patel et al., 2021). Extensive studies have been conducted during the last 5 years for the production of various bioproducts including compost (Lu et al., 2017; Jain et al., 2018; Ali et al., 2019; Mazumder et al., 2020), methane (Priya et al., 2018; Barua and Kalamdhad, 2019; Hudakorn and Sritrakul, 2020), hydrogen (Wazeri et al., 2018; Kumari and Das, 2019; Varanasi and Das, 2020), and ethanol (Bronzato et al., 2019; Kaur et al., 2019; Sunwoo et al., 2019). WH is also characterized by highest content of hemicellulose (up to 49.2% dry weight), compared to other aquatic plants and organic wastes (Zabed et al., 2016), which is a high potential for biofuel production. Researchers have been trying to use various mixtures and disposals for WH composting. On the other hand, different pretreatment technologies, and enzymatic hydrolysis and microbial fermentation were used to obtain biofuel from WH. These published researches are offering specific parameters to be taken into account, and their analysis to figure out the most relevant, and optimized conditions for biofertilizers and biofuels generation is highly recommended. So, in light of the last decade’s researches, this review provides a global vision, strategic solutions, and perspectives for all countries suffering from the widespread of this invasive plant. In this context, the authors have been working for 1 year on this review to explore an integrated vision leading to develop biofertilizers and biofuels after WH removal.

## 3 Methodology

**FIGURE 1:**
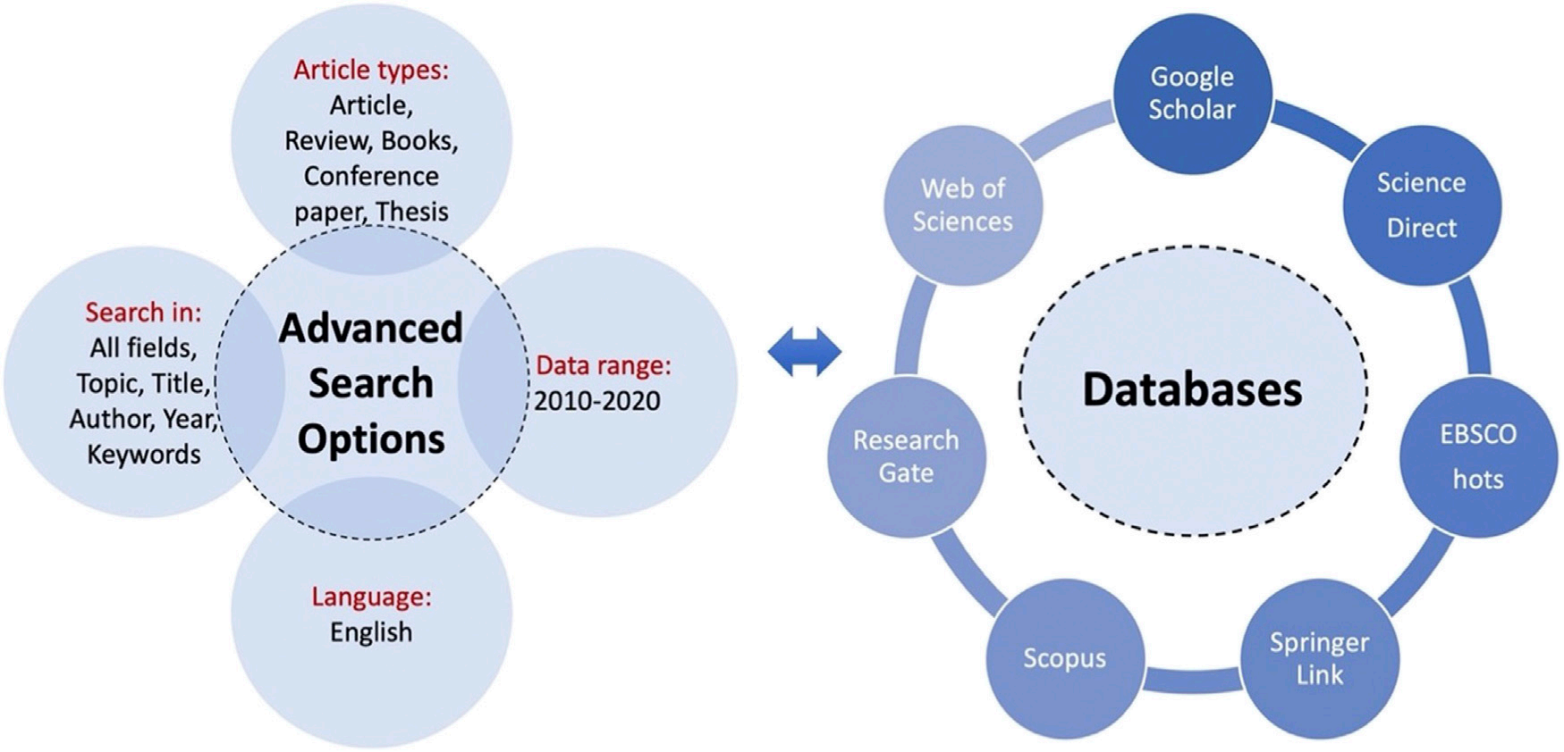
Databases and advanced search option.

This review is an exhaustive analysis aimed to synthetize and evaluate the bibliography content regarding the high spread of one of the top 10 worst weeds in the world and how it could be valorized through advanced biotechnology. Thereafter, this work is mainly focused on the promising treatment methods to produce bioenergy, and byproducts with high added value. The literature research was made using several databases (Figure 1). Thus, combinations of different keywords (WH, composting, anaerobic digestion, biofertilizers, methane, hydrogen, and bioethanol) related to this review’s topic were used. For each selected study, outcomes and date of publication were considered to evaluate further relevant studies.

Since the range of studies dealing with WH spread, its impacts, control approaches, and the appropriate ways for valorization through bioprocesses is very wide, some specific boundaries were selected (e.g., most affected area worldwide, last decade publications, and the pertinence of materials and methodology) to select paper’s relevance to this review.

**FIGURE 2:**
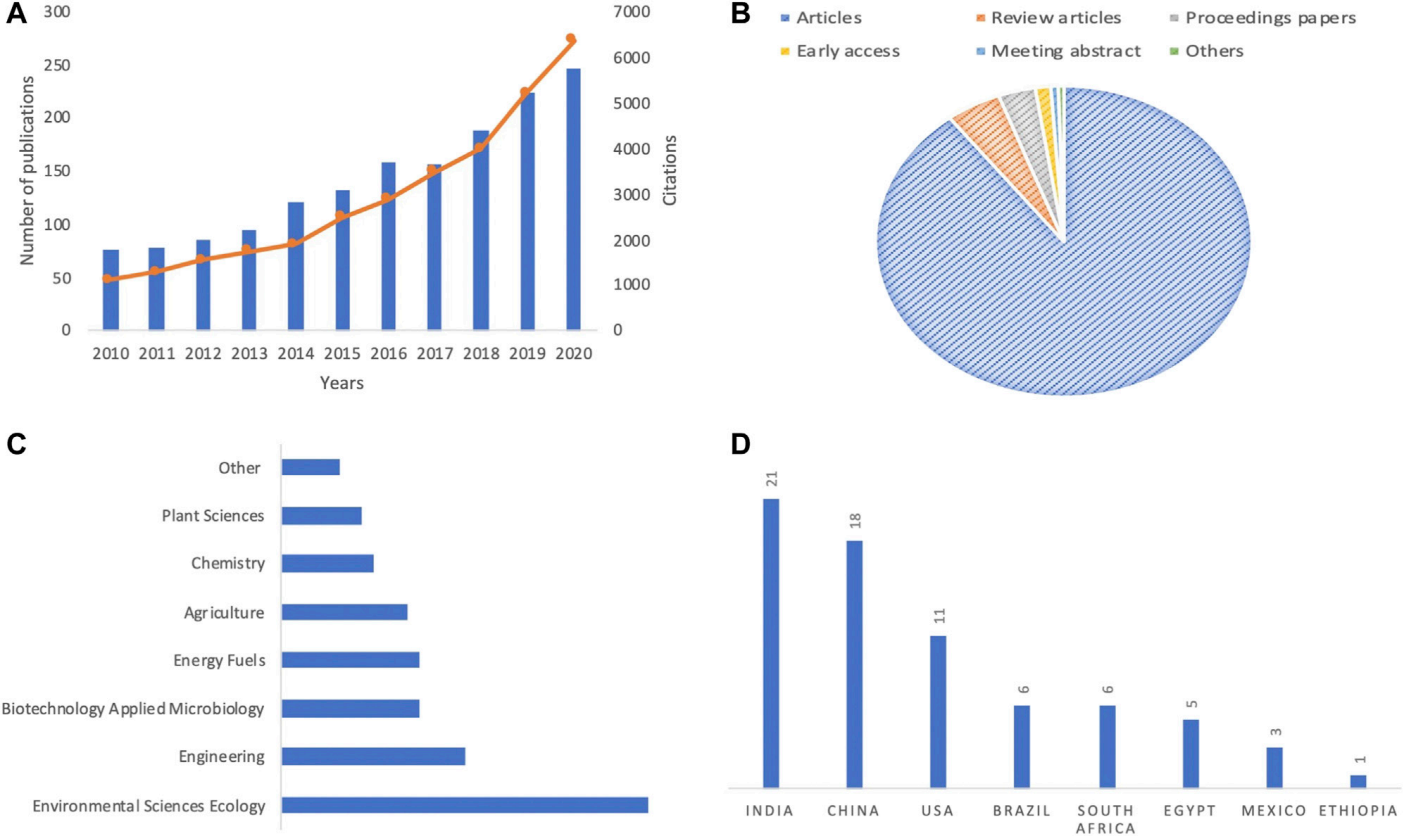
Overview of **(A)** total publications and citations, **(B)** percentage of published document per type, **(C)** the most relevant area **(D)** and countries of selected papers focused on WH spread, impacts, and management strategies.

Based on the number of publications and citations, Figure 2 a showed that an increasing interest in the valorization of WH is observed, within the last decade. Hence, more than 240 and 5,500 papers and citations were recorded during the last year, respectively. These scientific works are constituted by more than 90% of articles (Figure 2B) that are mostly related to some specific research fields, such as environmental sciences, engineering, and biotechnology applied microbiology and energy fuels (Figure 2C). On the other hand, special attention was given to these research works by researchers from India, China, the United States, and Brazil (Figure 2D). These findings are in accordance with a recent bibliometric analysis focused on research and sustainable use of WH (Basu et al., 2021). In the African continent, most of the published works are from South Africa, Egypt, Nigeria, Kenya, and Ethiopia. During the last 5 years, a high citation level was given to some specific scope of research related to bioenergy generation, wastewater treatment, and compost production from WH.

## 4 Worldwide Distribution, Social, Economic, and Environmental Impacts of Water Hyacinth

Various parameters are involved during the proliferation of water hyacinth. WH grows in fresh water with an optimum pH value, temperature, and total dissolved solids (TDS) ranging between 4–6, 1–40°C, and 37–183 mg/L, respectively. This invasive plant can’t tolerate high level of salinity,a salinity value above 10% is required for optimal growth, where water nutrient concentrations are high due to agricultural runoff, and insufficient wastewater treatment. Specific nutrient contents of nitrogen (0.5–20 mg/L) and phosphorus (0.02–3 mg/L) are the key elements for the WH spread. Briefly, WH is a very competitive species compared to the other aquatic species. It requires a wide range of environmental conditions for its proliferation. Its biological characteristics (reproduction, growth) and adaptation at a wide variety of habitats, including eutrophic and polluted waters with heavy metals, and make this plant an invasive species.

Generally, the bibliographic data related to WH spread showed that the covered area is mostly obtained after modeling and spatial coverage. However, the invaded areas are estimated after calibration of GPS points, shapefile creation in ArcMap and digitalization following GPS track points. GIS-based multi-criteria technique was also used to predict the WH hotspot invasion area (Dersseh et al., 2019). This approach remained suitable in terms of accuracy compared to the other ones. WH is present in more than 50 countries, where the economic costs of WH infestations have been estimated to be approximately $124 million per year (Wainger et al., 2018). In Africa, the costs may be as much as US$100 million annually (UNEP, 2006). WH is perfectly adapted to tropical regions in some African countries where it was observed for the first time in the Nile Delta in Egypt in 1800 (Dersseh et al., 2019). Actually, many African countries suffered from the high spread of this aquatic plant.

**FIGURE 3:**
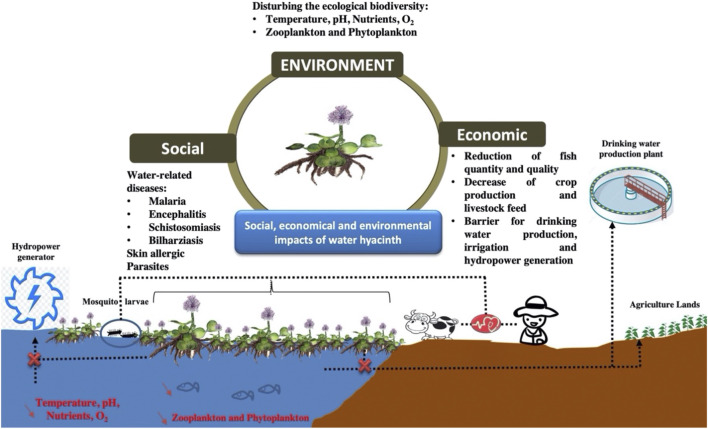
Social, economic, and environmental impacts of WH expansion.

WH spread is a major challenge causing social, economic, and environmental impacts (Figure 3). The social impacts of WH include an increase in the prevalence of malaria, encephalitis, and schistosomiasis (De Groote et al., 2003). It creates a conducive environment for the proliferation of mosquito larvae and the development of water-related diseases. On the other hand, it was reported that people engaged in its manual removal were usually suffering from skin allergies (Enyew et al., 2020). Cattle were also affected by leech and some other internal parasites (gut bloating and continuous diarrhea) caused by feeding water hyacinth. WH spread could also lead to the transportation of hazardous pollutants, such as plastic (Schreyers et al., 2021).

The economic impacts include mainly a reduction of fish quantity and quality. The proliferation of WH aggravates the decline of temperature, pH, nutrient content, and dissolved oxygen leading to fish death and disturbing the other constituents of the freshwater community (zooplankton and phytoplankton). On the other hand, the presence of this biomass on the water surface is creating a barrier for transporting water streams through canals (drinking water production and irrigation), and exploiting generators of hydropower plants.

## 5 Advanced Bioprocesses for Water Hyacinth Valorization

Converting WH into several products with high added value is recommended after its removal from water bodies.

The composition of WH must be well studied before testing one or a couple of valorization approaches. As shown in Table 1, WH has high content of cellulose, hemicellulose, and lignin. The lignin content in WH can reach up to 20%, the cellulose and hemicellulose could reach around 34 and 45%, respectively. WH is comprised of 85–91% water and 1–26% ash, 11–50% carbon, and 0.3–3% nitrogen. In addition, Table 1 indicated that the spatitemporal variation of growth conditions (climate and water composition) could affect the WH content (Dersseh et al., 2019; Dersseh et al., 2020). The composition of this plant favors its conversion into biofertilizer and bioenergy (biogas, biohydrogen, and bioethanol) and this was the aim of various experiences worldwide.

**TABLE 1:** The content of water hyacinth (fresh weight).

pH	Water content (%)	Cellulose (%)	Hemicellulose (%)	Lignin (%)	Ashe (%)	Carbon (%)	Nitrogen (%)	References
—	—	27.6	39.8	14.9	—	—	—	Pattra and Sittijunda, (2017)
5.58	88.8				28.7	49.3	1.1	Unpaprom et al. (2020)
—	—	24.5	34.1	8.6	1.5	—	—	Ruan et al. (2016)
—	—	18.2	29.3	2.8	1.2	—	—	Ma et al. (2010)
6.3	85	32.5	38.1	11		—	—	Bhattacharya et al. (2015)
—	—	17.3	24.7	1.1		—	—	Lay et al. (2013)
—	—	24.5	34.1	8.6	1.5	—	—	Ruan et al. (2016)
	—	25	35	10	20	—	3	Awasthi et al. (2013)
—	—	17.3	24.7	1.1	—	—	—	Chuang et al. (2011)
—	—	31.4	44.7	20	—	—	—	Das et al. (2016a)
—	90	24	30	16	20	38.4	2.9	Rathod et al. (2018)
	65.4	41.8	26.8	1.4	26.1	23	1.3	Ruenngam Jaruyanon, (2018)
6.5	91	29.4	32.2	5.2		11	0.3	Wazeri et al. (2018)
—	—	24.9	23.2	10.1	21.4	—	—	Zhao et al. (2014)
—	—	34.2	17.7	12.2	—	—	—	Ahn et al. (2012)
—	-	23.31	22.11	12.6	—	—	—	Xia et al. (2013)
—	86	35	35	15.5	—	—	—	Ganguly et al. (2013)
—	—	31.8	25.6	3.6	—	—	—	Yan et al. (2015)
—	—	18.1	28.2	7.1	—	—	—	Zhang et al. (2015)

### 5.1 Production of Compost From WH

Composting is an ecofriendly technology ables to convert a mixture of organic biomass to valuable by-product used as soil amendments (Ezzariai et al., 2018). WH was widely used as a co-substrate to produce compost with high added value and thereby recycl nutrients. As summarized in Table 2, composting efficiency, as well as the quality of WH-based compost are dependent on different parameters, i.e., initial mixture, moisture, pH, C/N ratio, quantity, and duration. Generally, WH is composted with the presence of other co-substrate (cattle manure, sawdust, green waste, cow dung, pig manure, peat, vegetable waste, food waste, and straw, etc.), because of its high moisture, nitrogen, and fibrous fraction that they would cause poor air diffusion and organic matter degradation (Jain et al., 2019).

**TABLE 2:** Production of WH-based compost.

Disposal of composting	Compost mixture	WH pretreatment	Initial moisture % (M), pH and C/N ratio	Compost quantities	WH proportion	Maximal temperature (°C)	Duration of composting (days)	Final C/N ratio	Studies outcomes	References
Rotary drum composter	Cattle manure + Sawdust + Green waste	WH was macerated to <1 cm	M = 70.39% pH = 6.9 C/N = 40	—	60%	—	20	12	-Compost from WH has no phytotoxicity risks on the seed germination of L. esculentum and B. oleracea -WH compost can be deployed for agriculture uses Future research is required for generating WH compost at large scale	Mazumder et al. (2020)
Pile composting	Vegetable waste + manure	—	M = 60% pH = 7 C/N = 30	2 m^3^	—	57	90	9	-WH composting is more efficient in the presence of some other substrates such as manure -The passive aeration is one of the best ways for WH composting	Martins et al. (2020)
Vermicomposting	Food wastes + Corncobs	—	pH = 7.9	—	50%	—	45	15.24	-WH has a greatest potential for producing compost using earthworms -Valorization of WH through vermicomposting is an important option to reduce its disposal costs and to produce biofertilizers for farming	Boonna et al. (2020)
Bags	Cacao pods	—	C/N = 44.57	—	50%	—	90	14.58	-The application of WH compost increased the soil microbial diversity -WH compost increased soybean nodulation and nitrogen fixation	Atere et al. (2020)
Pile composting	Cow dung	Autoclave (121 °C for 30 min)	pH = 7.2	—	—	—	50	12.3	-WH compost resulted a remarkable boost in the growth parameters of L.termis -Pretreated WH is recommanded for the formulation of compost to be used for the alleviation of salinity	Ali et al. (2019)
Rotary drum composter	Cow-dung + Sawdust + Biochar	—	M = 77% pH = 6.56 C/N = 29.1	150 kg	60%	56.9	20	21	-BC addition increased the moisture and VS reduction Essential nutrients are provided by BC addition -BC addition is recommended for WH composting (high temperature and organic matter degradation are recorded) -Some other studies are required to better understand the bioavailability of heavy metals in the presence of BC during WH composting	Jain et al. (2019)
Rotary drum composter	Cow dung + Sawdust	—	M = 55–80% pH = 6.75–7.25 C/N = 31	150 kg	60%	52	20	12	-Biochar prolonged the duration of the thermophilic stage, enhanced the organic matter degradation, and promote the nutritional quality -Biochar addition decreased metal content for composting of water hyacinth	Jain et al. (2018)
Pile composting	Cattle manure + Sawdust	WH was restricted to 1–2 cm	M = 81.45% pH = 7.8	150 kg	60%	55	30	—	-WH was found successful for composting at semi-industrial scale -Aeration and mixing have a huge impact on the thermophilic temperature and organic matter degradation	Varma et al. (2018)
Rotary drum reactor	Vegetable waste + Garden prune + Sawdust	—	M = 67% pH = 5.5 C/N = 20	550 L	10%	50	30	14	-Significant temperature was recorded during the process High GI was recorded -Composting was suggested as a suitable option to manage WH for the hilly region	Rich et al. (2018)
Pile composting	Straw	—	M = 90% pH = 8–9 C/N = 21	—	—	38	32	—	-WH may be used for the cultivation of mushrooms of Pleurotus -The best biological efficiencies are obtained in the presence of WH as a substrate	Mohammed and Mengist (2018)
Plastic containers	Pig manure + Peat	—	M = 60–70% pH = 7.5	50 L	33%	56	60	13	-Pig manure addition stimulated the composting of WH The composting of WH decrease the transformation of availability of Cu to residual Cu	Lu et al. (2017)
Rotary drum composter	Cow dung + Sawdust	WH was restricted to 1–2 cm	pH = 7	150 kg	60%	60	20		I-solated Bacillus Badius from WH compost was found as an efficient biosorbent to remove Pb (II) -Bioadsorption is depending on pH, rotational speed, temperature and biomass concentration This study could be extrapolated for other heavy metals	Vishan et al. (2017)
Bioreactor	Cow dung + Sawdust	—	pH = 8.9	—	—	—	84	10.1	-WH compost can be suitable for remediation experiment and leads to remove high concentrations of heavy metal (Mn, Fe, Zn, Cu and Cr)	Taiwo et al. (2016)
Vermicomposting	Cattle manure + Saw dust	Wh was restricted to <1 cm	M = 69% pH = 6.2 C/N = 31.7	1.5 kg	80%	—	45	12.3	-WH compost with high agronomic value is depending on some specific earthworm -Some earthworm species could accumulate heavy metals and some other ones are the best on account of biomass increment	Varma et al. (2016)
Pile composting	Cattle manure + Sawdust	WH was restricted to 1 cm	M = 60–83% pH = 5–6.7	150 kg	100–70%	58	30	—	-Heavy metals are bound with organic matter fractions (cattle manure and sawdust) during WH composting -The appropriate proportion of co-substrates affected significantly the available fraction of heavy metals	Singh and Kalamdhad (2016)

On the other hand, WH is well known for its high affinity to adsorb heavy metals, and other organic pollutants (Varma et al., 2016). So, composting could be a good way for treating this noxious plant, more particularly by adding biochar that was mostly used to reduce pollutants content below the inhibitory limit (Awasthi et al., 2017; Jain et al., 2018). Indeed, heavy metals and other organic pollutants did not show any inhibition effects, underlying the process efficiency. Vermicomposting was also suggested as one of the most promising biotechnologies due to the potential accumulation of pollutants by earthworms and then reducing their toxicological effects (Li et al., 2010; Zhang F. et al., 2016). The sequestration of pollutants is due to the mineralization and humification effect of earthworms leading to the conversion of heavy metals to an inert fraction (Zhang Q. et al., 2016). Elsewhere, the efficiency of various species of earthworm regarding WH composting at various nutrients levels and co-substrates is less studied.

According to our bibliographic analysis, many researchers adopted several composting disposals to produce quality-based compost from WH. Rotary drum composter, bioreactor and pile composting are the most used. Controlling basic parameters (moisture, pH, and C/N) before composting is mandatory to ensure microbial development and organic matter degradation. Despite its high macronutrients content (phosphorus, nitrogen, and potassium), WH cannot be composted alone and the presence of other co-substrates, as cited above, and is offering standard basic parameters before composting. Before starting composting, it is suggested that the mixture should have initial moisture, pH and C/N values ranging between 55–80%, 5–8, and 20–45, respectively. The mixtures with high WH ratio (more than 60%) showed significant temperature increase (up to 50°C). As indicated in Table 2, the duration of composting is varying from 20 to 90 days, which remains a non-significant duration to ensure compost quality, and maturity. Composting has also shown its efficiency to reduce lignin and cellulose content by about 10–40% (Jain et al., 2018). All these research resultsindicated positive effects on the physical, chemical, and biological stability of the final product.

WH composting leads to mitigation of high heavy metals (Taiwo et al., 2016) and the obtained WH-based compost has shown no phytotoxicity risks (Mazumder et al., 2020). Composting efficiency is highly correlated with the presence of some other co-substrates such as manure (Martins et al., 2020). The thermophilic stage is dependent on aeration and mixing rate (Varma et al., 2018), and its duration could be enhanced by biochar addition (Jain et al., 2018) and inoculation by some bacteria species and earthworms (Varma et al., 2016; Vishan et al., 2017; Boonna et al., 2020).

WH was used to conduct composting at a semi-industrial scale worldwide. For example, the plant was collected and composted, by a researchers team from the United States, and Mexico, to produce 50.5 m^3^ of compost and revenues of about1980 dollars (Montoya et al., 2013). More than 5,400 tons of WH were collected in Benin for composting and 3,200 tons of compost were produced. Many experiments were conducted in India, Sudan, Zimbabwe, Malawi, Ethiopia, and the Philippines indicating that composting at a large scale is the most used biotechnology for WH treatment and valorization. WH-based compost is a low-cost by-product that has shown high agronomic value, salinity alleviation and bioremediation. Nevertheless, some other studies are required to better understand the bioavailability of heavy metals according to WH ratio and composting duration, through composting and vermicomposting. In addition, inoculation and biological accelerators must be tested in order to enhance the biodegradation of this fibrous biomass during composting.

### 5.2 Water Hyacinth as a Source of Bioenergy

Biofuel production from WH involves physical, chemical, and biological pretreatment methods that are used to enhance the hydrolysis of carbohydrates to fermentable sugars, leading to improve methane, hydrogen, and ethanol production (Table 3). So, effective conversion of this biomass to biofuels is mostly associated with the adopted pretreatment approach. The potential changes in physical and chemical composition must be well studied to avoid any decrease biofuel in production. The selection of suitable approach to breakdown this complex structure and overcome all significant modifications is highly recommended (Rezania et al., 2019).

**TABLE 3:** Recent studies on WH pretreatment for biofuel production.

	Pretreatment	Conditions	Pretreatment effects	Studies outcomes	References
Methane production	Milling and thermal	Mechanical milling (1 mm) Dilution in deionized water (1:4) followed by thermal treatment at 80°C for 3 h Pretreatment inside hot air oven at 90°C for 1 h	The hydrolysis of WH was enhanced from 4 to 10–12% (sCOD/COD) No significant effect was observed on the methane yields	-Pretreatment duration of 30 min is sufficient for the solubilization of WH	Ferrer et al. (2010)
			Maximum biogas production of 197 mL over 11 days of incubation	-Hot oven drying of WH is recommended to increase the content of available simple soluble organic matter	Barua and Kalamdhad, (2019)
	Alkaline and enzymatic	NaOH with various concentrations (0.5, 1, 3, and 5 wt%) at 45°C for 24H + enzymatic hydrolysis (cellulase) at 45°C for 24 h + autoclave	NaOH pretreatment (3 wt%) and cellulase addition facilitate to produce glucose and xylose (143.4 ml –CH4/g-TVS and 51.7 ml-H2/g-TVS)	-WH leaves give the highest H2 and CH4 yields -Combined pretreatment (NaOH and cellulase) is recommended to increase H2 and CH4 yields	Cheng et al. (2010)
	Chemical	H2SO4 was used over different concentrations (0, 1, 2, 3, 4, and 5 %v/v) and residence time (0, 30, 45, and 60 min)	Chemical pretreatment changed cellulose and glucose content Pretreatment increased biogas production 131.45% compared to without pretreatment	-The best condition was H_2_SO_4_ concentration of 5% v/v with residence time of 60 min	Sarto et al. (2019)
		NaOH with various concentrations (1, 2, 3 and 4%) for 48 h	Morphological changes induced by NaOH are first noticeable after a pretreatment with 1% NaOH After 2% NaOH pretreatment, the outer layer was removed, and the cell cluster was broken into small species The cell wall of each cell was exposed with 3 and 4% NaOH pretreatment	-After the alkali pretreatment, the structure of WH was changed expressively	Unpaprom et al. (2020)
	Physical, chemical, and biological	Autoclave (121°C for 30 min) Microbial consortium of fungi and bacteria Alkali pretreatment with NaOH (2.5%) and NH4OH	Pretreated WH indicated high yield biogas production (150 ml CH4/g VS) on the 21^st^ day	-Autoclave pretreatment enhances the biogas production	Ali et al. (2019)
Bio-hydrogen production	Chemical, physical, and biological	NaOH (0.2wt%) for 24 h Microwave (150, 190, 210, and 230°C) for 30 min Cellulase addition at different dosages (0, 0.5, 1, 2.5, 10, 25, 50, and 100 wt%) at 190°C for 10 min	Microwave pretreatment is more effective from 5 to 10 min After enzymatic hydrolysis, reducing sugars increased to 0.296 g/gTVS	-Enzymatic digestibility is more enhanced after microwave and alkaline pretreatment	Lin et al. (2015)
		Stream heating: 112°C for 15 min Microwave: 1% NaOH, 45°C, 120 rpm, 24 h, (420w, 1 min) Enzymatic hydrolysis: Cellulase + CaCl2 (48 h, 120 rpm)	Reducing sugar yields from steam heating and microwave heating/alkali pretreatment were 0.66–0.78 and 0.47–0.54 g/100 g TVS In steam heating and enzymatic hydrolysis pretreatment, the produced reducing sugar yields for 10e40 g/l of WH were 6.15, 7.34, 7.78, and 8.86 g/100 g TVS respectively	-Steam heating, microwave heating/alkali and enzymatic hydrolysis are promising methods to promote the production of reducing sugar and hydrogen from WH	Su et al. (2010)
		Microwave: 1% H2SO4, 140°C, 15min Enzymatic hydrolysis: Cellulase + CaCl2, 45°C, 120 rpm, 120 h	After microwave-acid pretreatment, reducing sugar yields of 49.4 g/100 g was obtained	-This study observed H2 yield of about 134.9 lK/g TVS -Detoxification and domestication increased H2 yield production	Cheng et al. (2015)
	Chemical	2.5% H2O2 and 1% NaOH Mixture was kept at 50°C for 3 different time period (30, 60, 90, 120, and 150 min)	Combined H2O2 and alkali treatment reduced lignin, cellulose and hemicellulose contents by 85, 4.75, and 22.33% (w/w)	-Applied pretreatment are effective to remove lignin -Pretreatment and WH addition gave maximum production of H2 and CH4 from sugarcane bagasse	Kumari and Das, (2019)
		Acidic pretreatment (H2SO4, 1.3% (v/v), pH 8.1, 30°C)	Acidic pretreatment leads to produce 182.7 mmol H2/L	-Heat-treated anaerobic digestion is a promising way for H2 production -The main H2 production process is butyrate fermentation	Pattra and Sittijunda, (2017)
Ethanol production	Physical and biological	Microwave heating (10 ml of H2SO4 (0–2%), 2.45 GHz, 120–200°C, 0.1–3 MPa, 5–45 min) + Enzymatic hydrolysis	Residual solid biomass decreased from 43 to 23% Cellulose decreased from 80 to 38% Sugar yield obtained after enzymatic hydrolysis was 48.3 g/100 g hyacinth	-Microwave-acid pretreatment enhanced enzymatic saccharification of WH	Song et al. (2017)
		Steam explosion (190°C for 1–10 min) Enzymatic hydrolysis (*Trichoderma harzianum, Saccharomyces cerevisiae*)	Maximum reducing sugar is about 15.5 g/L The efficiency of enzymatic hydrolysis is 0.51 reducing sugars per Gram of WH Ethanol yield is about 0.23 g/g of dry matter	-This combination is a promising option of reducing sugars from WH -Steam explosion allows high efficiency of enzymatic hydrolysis	Figueroa-Torres et al. (2020)
	Physical, chemical, and biological	Hyper-thermal acid hydrolysis: H2SO4 (100–400 mM), temperature (140–200°C), time (5–30 min) Enzymatic saccharification*: Cellic CTec2 + Viscozyme L*	Highest monosaccharide production (Ep = 45%) is obtained at 8% slurry, 200 mM H2SO4, 160°C and 20 min A maximum monosaccharide content of 41.7 g/L was obtained when an enzyme mixture of Cellic CTec 2 and Viscozyme L	-HT acid hydrolysis and enzymatic saccharification enhanced monosaccharide production -Fermentation with adapted P. stipitis and C. lusitaniae produced higher ethanol concentrations from xylose	Sunwoo et al. (2019)
	Chemical and biological	0.5% NaOH (121°C for 30 min) and enzymatic hydrolysis	High reduction of lignin 46–58%	-Alkali-pretreated WH is a promising approach for ethanol production (8.4 g/L)	Narra et al. (2017)
		2.5% H2SO4 at 121°C for 30min Adjustment of pH (5–10), activated charcoal addition, shaking for 60 min at 55°C and filtration (0.2 mm) Saccharification by enzyme addition (*Sphingobacterium sp ksn*)	Maximum xylose (18.32 and 21.95 g/L) was obtained using H2SO4 at 2.5%	-WH is a suitable substrate for ethanol and xylitol production	Shankar et al. (2020)
			0.51 and 0.19 g L−1ofpolyphenols were left in the hemicellulosic hydrolysate of BL and WHL Through Sphingo bacterium sp. Ksn treatment, 15–18 g/L of glucose was produced Maximum xylitol obtained was about 8–10 g/L		
		1% H2SO4 (AC) and 4% NaOH (AK), 1 h, 100°C *Phanerochaete chrysosporium* (MB) was used before fermentation	MB decreased total dry matter by 26.67% MB + AK pretreatment decreased lignin content by 33.3% The reducing sugars could achieve 430.66 mg/g and 402.10 mg/g after MB + AC and MB + AK pretreatment After MB + AC and MB + AK pretreatment, the production of glucose achieved 164.11 mg/g and 182.35 mg/g	-WH for bioethanol production seemed to be a sustainable option -MB + AC pretreatment is a promising approach for reducing sugars and then improving bioethanol production	Zhang et al. (2018)

In this section, the most used pretreatment approaches will be described as well as the experimental conditions leading to produce bioenergy through anaerobic digestion and microbial fermentation.

#### 5.2.1 Pretreatment Methods

As summarized in Table 3, all studies have concluded that pretreatment is recommended for enhancing methane production. Therefore, physical and chemical pretreatments are the most applied techniques. Fresh WH must be washed, air dried, and chopped to small pieces (3 cm at least). After that, WH is oven dried (60–105°C for 1–3 days) and then mechanically ground (particle size of about 1 mm is recommended). Thermal pretreatment at 80–90°C for 1–3 h, indicated that less than 1 h treatment is more sufficient to obtain a maximum biogas production of 197 ml per 11 days of incubation (Ferrer et al., 2010; Barua and Kalamdhad, 2019). Chemical pretreatment has been mainly conducted by NaOH and H_2_SO_4_ at concentrations varying from 0.5 to 5%, and a residence time from 0 to 60 min (Cheng et al., 2010; Ali et al., 2019; Sarto et al., 2019; Unpaprom et al., 2020). Autoclaving (45–121°C for 30–60 min) and enzymatic hydrolysis (cellulase or some selected microbial consortium) were conducted after the acidic/alkaline pretreatment. This combination has led to induce morphological changes in WH texture, to facilitate glucose and xylose production and to increase the production of methane (until 150 ml CH_4_/g VS per 20 days of incubation) (Ali et al., 2019). These results indicated that a combined pretreatment (chemical, enzymatic) is a promising approach to increase methane production from WH.

The chemical pretreatment (NaOH, H_2_O_2_, and H_2_SO_4_) was tested at different concentrations (0.2–2.5%) to enhance hydrogen generation from WH (Table 3). In spite of its ability to reduce lignin, cellulose and hemicellulose, and the application of chemical treatment alone is less effective compared to the combined one. This limitation could be mitigated by detoxification after acidic/alkaline treatment (Cheng et al., 2015). A combination of chemical, physical and biological pretreatments is sufficient for lignin removal, reducing sugar, and then increasing the enzymatic digestibility for hydrogen production (Cheng et al., 2015; Lin et al., 2015; Song et al., 2017). Microwave was mostly used as a physical pretreatment before the enzymatic hydrolysis (cellulase), and this technique offers a significant decrease in solid biomass (up to 20%) and sugars yields (until 50 g/100 g).

Ionic liquid pretreatment based on imidazolium-, pyridinium-, ammonium-, and phosphonium-based cations, associated with alkyl or allyl side chains coupled to anions, such as chloride, acetate, and phosphonate, have been widely used for the pretreatment of lignocellulosic biomass (Wang et al., 2017). This technique is well known for improving the saccharification efficiency and biomass digestibility (Fu and Mazza, 2011; Haykir et al., 2013). Ionic liquid was recentlty used for WH pretreatment, by dissolving the ionic liquid with the sample, and followed by incubation at 100–150°C for 10–120 min. The obtained extract is mainly filtred, and the residue is washed deeply before drying at low temperatures (Guragain et al., 2011; Gao et al., 2013; Chang et al., 2017). The hydrolysis and sugar reduction increased significantly (2–3 times) by ionic treatment compared to acidic or alkaline pretreatment (Guragain et al., 2011). Powder X-ray Diffraction, FTIR and Scanning Electron Microscopy indicated high crystallinity index, significant removal of lignin, and the alteration of the WH structure after ionic liquid pretreatment (Singh et al., 2018). For enhancing biofuel production form WH, this technique could be also combined with various physical and chemical pretreatment methods.

Ethanol production from WH involves chemical pretreatment, enzymatic hydrolysis (saccharification), and fermentation. These 3 steps lead to the break down of the fibrous content to make it more susceptible to hydrolysis, conversion to of cellulose and hemicellulose into sugar monomers, and finally the fermentation of sugars to ethanol (Thi et al., 2017). Acidic pretreatment is the most effective way for dissolving hemicellulose and retaining most of the cellulose. High temperatures (100–200°C) and concentrations of H_2_SO_4_ and NaOH, varying from 0.5 to 2.5% are usually applied. The enzymatic hydrolysis (*Cellic CTec2, Viscozyme L, Sphingobacterium* sp *ksn*, and *Trichoderma harzianum*) indicated that saccharification is necessary for high sugar reduction. During fermentation, addition of conventional yeast (*Saccharomyces cerevisiae* for example) is highly critical forthe microbial conversion of reducing sugars. Our bibliographic analysis indicated that high ethanol production from WH is associated with low lignin content before the fermentation. So, chemical pretreatment and enzymatic hydrolysis must be well controlled to ensure more than 60% of lignin reduction.

#### 5.2.2 Progress on the WH Valorization for Methane, Hydrogen, and Ethanol Production

The production of biofuels from WH is summarized in Table 4. Previous studies on methane production from WH by anaerobic digestion showed high potential yields. Generally, the high potential yield of methane is related to pH, suitable temperature, and optimized ratio between inoculum and substrate (Uddin et al., 2021; Chew et al., 2021; Ilo et al., 2021). In addition, several studies mentioned that pretreatment is beneficial for enhancing methane production (Suthar et al., 2022). Anaerobic digestion on batch or semi-continuous mode is the most used disposal. Different inoculation conditions were tested (sludge, cow dung, and swine dung, etc.), under mesophilic temperature for an incubation durations from 2 to 180 days. As indicated in Table 4, methane was recovered from WH in the range of 193–400 ml CH_4_/g VS by using substrate concentration varying from 1 to 48 g/L. Co-digestion has the potential to increase methane yield and reduce the duration of incubation (Unpaprom et al., 2020). Some studies indicated that the use of food waste, cow dung and some other organic waste decreased the incubation to 30 days (Priya et al., 2018; Kunatsa et al., 2020). In some cases, microbial pretreatment was used to generate methane from WH. This microbial community leads to solubilize WH into carbohydrates which are converted by acidogenic, acetogenic, and methanogenic microorganisms for the generation of methane (Barua and Kalamdhad, 2017). Generally, the composition of WH, inoculation conditions, incubation time and temperature are the most determinant factors influencing methane production from WH. Hence, optimization work is almost suggested by conducting biochemical methane potential tests before starting anaerobic digestion in batch or semi-continuous mode.

**TABLE 4:** Biofuel production from WH through anaerobic digestion and microbial fermentation.

Bioprocess	Inoculum/co-substrates	Pretreatment	Temperature (°C)	Initial pH value	Treatment duration (days)	Substrate concentration	Outcome	Potential yield	Reference
Batch Anaerobic digestion	Digested sewage sludge	-Milled (1 mm)-Thermal pretreatment at 80°C	35–55	6	20	5–10 g VVS/L	Methane	3–6.5 L CH4/kg COD/day	Ferrer et al. (2010)
	—-								
		-Microbial pretreatment (*Citrobacter werkmanii* VKVVG4)	30	7	50	—	Methane	0.2 CH4/kg COD/day	Barua and Kalamdhad, (2018)
	Activated sludge + Food waste	Sun drying	27	6.3	15	-	Methane	150–400 ml CH4/g VS	Priya et al. (2018)
	Cow dung	—	37	7	30	3:1 ratio	Methane	63%	Bhui et al. (2018)
	Cow dung	Autoclave Biological and Alkaline pretreatment	37	7	50	—	Methane	150 ml CH4/g VS	Ali et al. (2019)
	—	—	35	6.8	60	—	Methane	237.37 L CH4/kg VS added	Hudakorn Sritrakul, (2020)
	Domestic sludge	Pretreatment with NaOH and cellulase (45°C for 24 h)	25	6	2	1 g/L	Methane	143.4 ml-CH4/g-TVS	Cheng et al. (2010)
	Sludge	—	37	7.5	20	20 g/L	Methane	58.9 ml/d	Varanasi et al. (2018)
	Cow dung	Hot air oven (1 h at 90°C)	30	7	50	1:5 ratio	Methane	193 ml CH4/g VS	Barua and Kalamdhad, (2017)
	Sludge	Chemical pretreatment (H_2_SO_4_)	37	7	90	—	Methane	64.38%	Sarto et al. (2019)
	Cow dung + wastepaper	Sun-dry for a period of 30 days	37	7	30	—	Methane	60%	Yusuf and Ify, (2011)
	Solid waste + Cow dung	—	30	7	30	53:27 ratio	Methane	48.7%	Kunatsa et al. (2020)
	Ruminal slaughterhouse waste	—	37	8.2	60	7 g/L	Methane	69%	Omondi et al. (2019)
	Food wastes	—	37	7	45	8:3 ratio	Methane	298.83 ml/g VS	Zala et al. (2020)
	Swine dung	Alkaline pretreatment (NaOH)	35	6.9	45	25 g/L	Methane	68.89% CH4	Unpaprom et al. (2020)
	Pig slurry	—	62.5	7	33	47.8 g/L	Methane	24.4 mmol/CH4/L/d	Chuang et al. (2011)
	Hydrogen-producing bacteria	Microwave, enzymatic hydrolysis and Alkali pretreatment	35	8	6	50 g/L	Methane	65 mL/g	Lin et al. (2015)
Continuous anaerobic digestion	Food wastes + Cow dung	Pulverization + Hot air oven (1 h at 90°C)	35	7	70	—	Methane	63.67%	Barua and Kalamdhad, (2019)
	Sugarcane bagasse	—	37	6.5	14	1:2 ratio	Methane	142 ml/g COD	Kumari and Das, (2019)
	Cow manure + kitchen waste	Alkaline pretreatment (NaOH)	37	7	180	1:1 ratio	Methane	65%	Tasnim et al. (2017)
Batch Fermentation	Sugarcane bagasse	Alkaline	37	6.5	1	10 g/L	Hydrogen	303 ml/g COD	Kumari and Das, (2019)
	Mixed culture bacteria + inoculum sludge	—	55	6	3	5 g/L	Hydrogen	67.1 ml/g	Wazeri et al. (2018)
	Hydrogen producing bacteria	Microwave + Enzymatic hydrolysis	37	6	1	10 g/L	Hydrogen	48.6 ml/g	Song et al. (2017)
	Sludge	—	35	6	—	5 g/L	Hydrogen	119.6 ml/g	Elsamadony and Tawfik (2018)
	Hydrogen producing bacteria + nutrients	Microwave, enzymatic hydrolysis and Alkali pretreatment	35	8	6	50 g/L	Hydrogen	180 ml/g	Lin et al. (2015)
	Sludge	Acidic pretreatment (H2SO4)	30	5.81	17	4.06 g/L	Hydrogen	182.7 mmol H2/L	Pattra and Sittijunda, (2017)
Dark fermentation + photo-fermentation	Electroactive culture medium + nutrients	—	25	7	2	20 g/L	Hydrogen	67.69 L H2/kg COD	Varnasi and Das, (2020)
	Anaerobic activated sludge	Microwave and alkali pretreatment	35	7	2	10 g/L	Hydrogen	596.1 ml/g	Su et al. (2010)
Dark fermentation	Hydrogen producing bacteria + nutrients	Microwave-acidic-enzymatic pretreatment	35	6	2	25 g/L	Hydrogen	134.9 ml/g	Cheng et al. (2015)
	Hydrogen producing bacteria + nutrients	—	37	6.5	0.5	20 g/L	Hydrogen	900 ml/L	Varanasi et al. (2018)
	Pig slurry	—	62.5	7	33	47.8 g/L	Hydrogen	221.3 mmol H2/L/d	Chuang et al. (2011)
Batch Fermentation	Slurry	Hyper-thermal acid hydrolysis + enzymatic saccharification	30	5	3	8 g/L	Ethanol	22.7 g/L	Sunwoo et al. (2019)
	*Saccharomyces cerevisiae* strain + Mixed microbial consortia	Hydrothermal treatment	37	7	15	5 g/L	Ethanol	21 g/L	Kaur et al. (2019)
	Nutrients + *Pichia stipitis*	Alkali and enzymatic pretreatment	28	5	6	5 g/L	Ethanol	3.2 g/L	Kumari et al. (2014)
	—	Acid pretreatment	50	5.5	1	10 g/L	Ethanol	13.6 g/L	Das et al. (2016b)
	Nutrients	Alkali and enzymatic pretreatment	30	5	1	25.8 g/L	Ethanol	4.13 g/L	Ganguly et al. (2013)
	—	Alkali and enzymatic	30	5.5	8	5 g/L	Ethanol	8.04 g/L	Narra et al. (2017)
	Banana waste + nutrients	Acid and enzymatic treatment	30	5	2.5	20 g/L	Ethanol	8.1 g/L	Shankar et al. (2020)
	Nutrients	Microbial + Acid + Alkaline	30	7	1.5	6 g/L	Ethanol	1.4 g/L	Zhang et al. (2018)
	Nutrients	Alkaline and enzymatic	30	5	1.5	10.4 g/L	Ethanol	8.2 g/L	Das et al. (2015)
	Nutrients	Autohydrolysis, acid hydrolysis, peroxide hydrolysis and enzymatic hydrolysis	32	5	3	25 g/L	Ethanol	0.066 ml/g	Bronzato et al. (2019)
	Nutrients	Acid pretreatment	37	7	2	10 g/L	Ethanol	6.2 g/L	Rezania et al. (2019)
	Nutrients	Steam explosion and enzymatic hydrolysis	30	5.5	3	10 g/L	Ethanol	7.13 g/L	Figueroa-Torres et al. (2020)

Degradation of WH at pH values of 6−7 and in the presence of mixed bacterial cultures (*Clostridium*, *Enterobacter, Rhodobacter,* and *Rhodopseudomonas*) leads to a quick delignification and enhances hydrogen production. Previous researches have indicated that high cellulase and xylanase enzyme activities are obtained by sodium bicarbonate or sodium chlorite addition, which enhance hydrogen production (Varnasi and Das, 2020). Hydrogen production from WH is mostly conducted in batch fermentation, dark fermentation and photo-fermentation. Dark fermentation and photo-fermentation could be coupled since non-fermentable acetate or butyrate would be used for further hydrogen generation (Chookaew et al., 2014; Khongkliang et al., 2017). Inoculation is mainly based on a mixture of hydrogen producing microorganism in the presence of nutrients. In some cases, the medium was also inoculated with sludge or pig manure. Pretreatment is highly required and it is carried out by alkaline, microwave and enzymatic hydrolysis. The highest hydrogen yields were obtained by combining microwave, acid, and enzymatic hydrolysis. At high temperature and acidic conditions, it is suggested to use activated carbon to remove fermentation inhibitors (acetic acid, furfural, and phenolic compounds) in hydrolysates, which are used to acclimatize hydrogen producing microorganisms (Cheng et al., 2015). This approach is highly recommended because it increases hydrogen yield from 104 to 134 ml/g TVS.

Elsewhere, high ethanol yield is obtained at an organic load of 8 g/L and an incubation time of 7 days. Microbial fermentation is mostly conducted at mesophilic temperature (Zhang F. et al., 2016). The selected species must be able to convert monosaccharides to ethanol and tolerates the presence of some potential inhibitors in the hydrolysate (Kumar et al., 2009). Among the most used yeast species that are known by their hexose, pentose and xylose fermentation are *Saccharomyces cerevisiae,* and *Pichia stipitis* (Kumar et al., 2009). The use of these species in combination could improve ethanol production from WH.

The analysis of the main findings of this section indicated the most outputs that are summarized hereafter:

⁃ To produce methane, WH is co-digested (sludge, food waste, cow dung, selected bacteria, and fungi) under temperatures ranging from 27 to 62°C and pH around neutrality. More than 70% of the published articles showed that WH is mainly pretreated using acidic, alkaline or milling approaches;
⁃ High potential yield of methane is mainly obtained after pretreatment steps. It seems difficult to compare the published potential yield of methane and the other major outcomes coming from anaerobic digestion, regarding the heterogenous used units. Standard units must be used for future works to better improve the treatment conditions;
⁃ Dark fermentation is the optimal process to produce bio-hydrogen and its coupling with photo-fermentation could enhance the potential yield;
⁃ WH could be also used to produce bio-ethanol in the presence of an inoculum;
⁃ Batch fermentation is mainly used after biomass pretreatment at some specific conditions of temperature and pH for ethanol production.

### 5.3 Road Map for Future Valorization Approaches to Produce Bioenergy From WH

To establish a road map for the future work which will be focused on the valorization of WH based on anaerobic processes, the summarized data on Table 4 were analyzed based on Multi Factorial Analysis (MFA) in order 1) to visualize the most relevant variables that control the bioprocess and *2*) explore optimized values to be considered before producing methane, hydrogen or ethanol from WH.

As shown in Table 4, potential yield is expressed by different units and they cannot be used for all anaerobic processes to establish the same statistical analysis. However, bioprocesses leading to the production of ethanol and methane/hydrogen have been treated separately.

**FIGURE 4:**
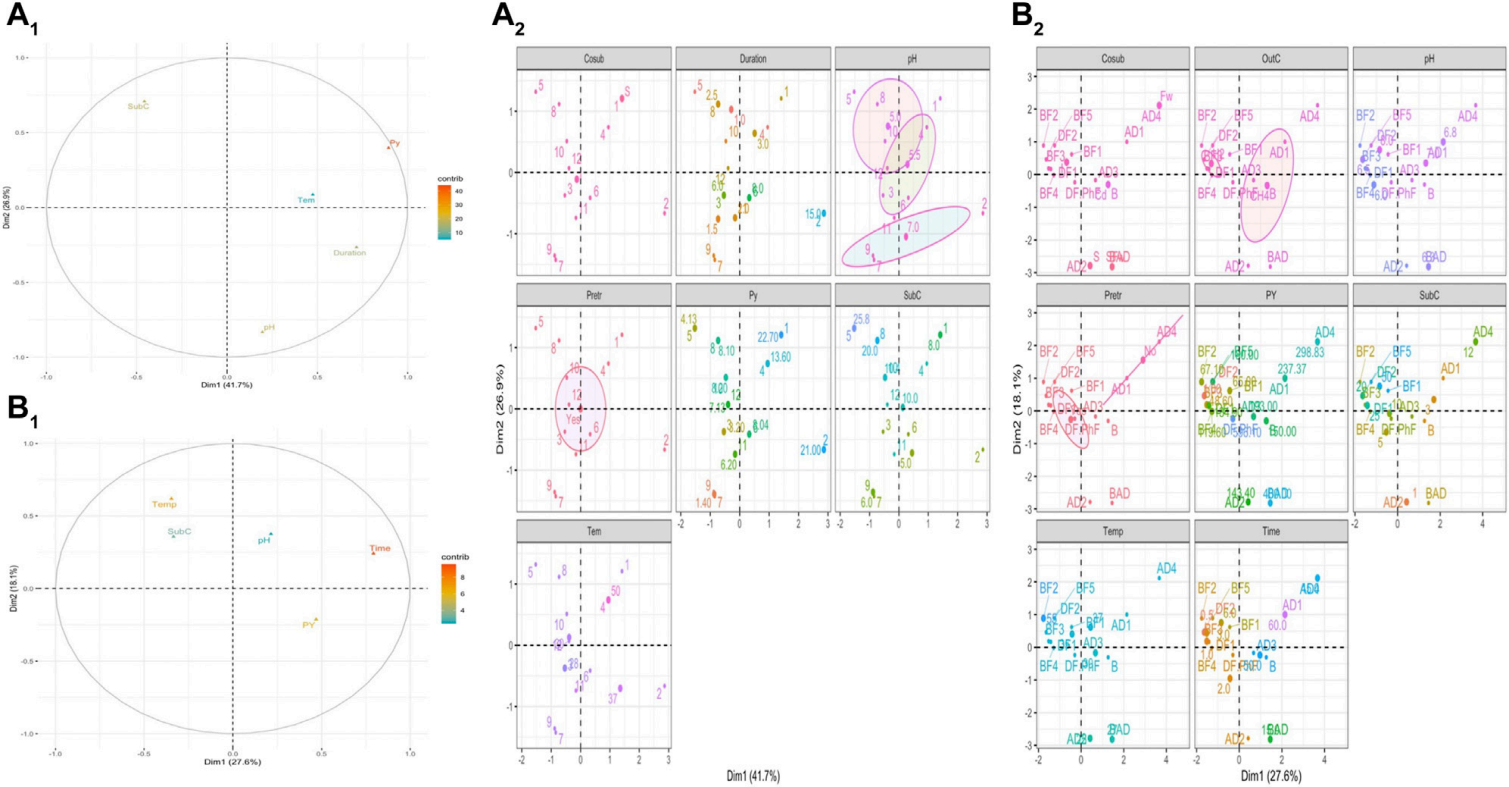
Multi factorial analysis of the summarized data in the Table 2 to investigate optimal conditions for **(A)** ethanol, **(B)** methane and hydrogen production. SubC: substrate concentration; Py: potential yield; temp: temperature.

⁃ The analysis of quantitative variables—MFA (Figure 4a_1_) showed that the substrate concentration is the key element that contributes mainly to MFA construction, indicating that this parameter controls the other ones and then the potential of ethanol production.
⁃ MFA factor map (Figure 4a_2_) shows that the high potential yield of ethanol production is related to the use of inoculum, incubation time of 2.5 days, pH 5.5, the application of pretreatment, and substrate concentration of 20–25 g/L and incubation temperature of 30°C.
⁃ Time of incubation and temperature are the major variables that control processes leading to production of methane and hydrogen (Figure 4b_1_).
⁃ The production of high yields of methane and hydrogen required the use of an inoculum, an incubation time of 50 days, pH value of 6.8, application of pretreatment, substrate concentration of 12 g/L and incubation temperature around 50°C.
⁃ To produce methane and hydrogen, continuous anaerobic digestion and dark fermentation are the most suitable bioprocesses, respectively (Figure 4b_2_).

## 6 Conclusion

WH spread is a major challenge causing social, economic and environmental impacts. Otherwise, converting this biomass, after its removal, and into several valuable products is well recommended. Composting is the most promising and recommended technology to be implemented at a large-scale. Nevertheless, some other studies are required to better understand the bioavailability of heavy metals according to WH ratio and composting duration. In addition, inoculation and biological accelerators must be tested in order to enhance the biodegradation of this fibrous biomass during composting and vermicomposting.

WH is considered a valuable plant for biofuel production. For methane production, WH is co-digested under mesophilic temperature. High methane yield is obtained after pretreatment (milling, acidic, and alkaline). On the other hand, dark fermentation is well recommended to produce hydrogen from WH. Combined pretreatment (physical-chemical-biological) is highly recommendedto increase bioethanol production from WH. Ionic liquid pretreatment is a promising method that opens up an attractive and green alternative route for WH pretreatment.

The most used techniques of WH removal (physical, chemical, and biological) showed limited effects for controlling WH spread. This review indicated that massive valorization of WH through advanced bioprocesses could be a promising strategy. After the physical removal of WH, countries that are suffering from this invasive plant could generate high incomes through its valorization, and especially at large scale. Based on an important database and advanced statistical analysis, this paper explored the most relevant conditions for generating biofertilzers, and bioenergy. Unfortunately, the scale-up of these bioprocesses is less encouraged and few experiences hase been listed. The policymaker should encourage implementing industrial pilot for WH valorization through aerobic, anaerobic or hybrid processes. In addition, increasing the support of such techniques will be a remarkable solution, and especially within the framework of a circular economy.
